# Pyrosequencing analysis of the human microbiota of healthy Chinese undergraduates

**DOI:** 10.1186/1471-2164-14-390

**Published:** 2013-06-10

**Authors:** Zongxin Ling, Xia Liu, Yueqiu Luo, Li Yuan, Karen E Nelson, Yuezhu Wang, Charlie Xiang, Lanjuan Li

**Affiliations:** 1 State Key Laboratory for Diagnosis and Treatment of Infectious Diseases, the First Affiliated Hospital, College of Medicine, Zhejiang University, Hangzhou, Zhejiang, 310003, China; 2 Department of Intensive Care Unit, the First Affiliated Hospital, College of Medicine, Zhejiang University, Hangzhou, Zhejiang, 310003, China; 3 J. Craig Venter Institute, Rockville, Maryland, 20850, USA; 4 Chinese National Human Genome Center at Shanghai, Shanghai, 201203, China

**Keywords:** Healthy microbiota, Saliva, Nasopharynx, Feces, Dominant hand, Pyrosequencing, Core microbiome

## Abstract

**Background:**

Elucidating the biogeography of bacterial communities on the human body is critical for establishing healthy baselines from which to detect differences associated with disease; however, little is known about the baseline bacterial profiles from various human habitats of healthy Chinese undergraduates.

**Results:**

Using parallel barcoded 454 pyrosequencing targeting on the 16S rRNA gene V3 region, the bacterial diversity of the nasopharynx, saliva, dominant hands, and feces were investigated from 10 healthy Chinese junior boarding undergraduates at Zhejiang University. The participants were 21–24 years of age with a body mass index (BMI) < 24 kg/m^2^. A total of 156,717 high-quality pyrosequencing reads were obtained for evaluating bacterial diversity, which represented 29,887 unique phylotypes. The overall taxonomic distribution of the 16S rRNA gene-based amplicons demonstrated that these 4 habitats of the human body harbored distinct microbiota and could be divided into different clusters according to anatomic site, while the established patterns of bacterial diversity followed the human body habitat (feces, hands, saliva, and nasopharynx). Although significant inter-individual variation was observed, the healthy microbiota still shared a large number of phylotypes in each habitat, but not among the four habitats, indicating that a core microbiome existed in each healthy habitat. The vast majority of sequences from these different habitats were classified into different taxonmies that became the predominant bacteria of the healthy microbiota.

**Conclusions:**

We first established the framework of microbial communities from four healthy human habitats of the same participants with similar living environments for the Chinese undergraduates. Our data represent an important step for determining the diversity of Chinese healthy microbiota, and can be used for more large-scale studies that focus on the interactions between healthy and diseases states for young Chinese adults in the same age range.

## Background

An enormous number of microorganisms, the vast majority of which are bacterial species, are known to colonize and form complex communities, or microbiota, at various sites within the human body [1,2]. Microbial cells that thrive on and within the human body are approximately 10 times more numerous than our own cells and contain, in aggregate, approximately 100 times more genes, leading to the suggestion that humans and our microbial symbionts be considered “supraorganisms” [3]. The microbiota can be viewed as a forgotten “organ” exquisitely tuned to our physiology that performs functions that we have not had to evolve on our own [4]. A growing body of evidence suggests that the composition and function of microbiota in different human body habitats, including nasal passages, oral cavities, skin, gastrointestinal tract, and urogenital tract, play vital roles in human development, physiology, immunity, and nutrition [1,5-15]. With the advent of molecular techniques, especially the development of next-generation high-throughput sequencing techniques, more and more bacterial phylotypes, which were undiscovered by traditional microbiological techniques, were found in these habitats of the human body. Prior studies have shown that the taxonomic composition of the microbial community may affect the propensity to develop obesity, inflammatory bowel disease, type 1 diabetes, cardiovascular diseases, bacterial vaginosis, dental caries and so on [7,16-22]. Studies of healthy individuals, including the Chinese population, have focused on particular body habitats, such as the gastrointestinal tract, nasopharynx, skin, and oral cavity, using high-throughput sequencing techniques [16,20-28], and have revealed microbial communities with significant intra- and inter-individual variations [27,29]. However, the five microbial habitats in the human body mentioned above are not isolated from one another; instead, each person comprises a complex, yet interconnected landscape consisting of many body habitats harboring distinctive microbiota [30]. Recently, the Human Microbiome Project (HMP) has analyzed the largest cohort and set of distinct, clinically relevant body habitats from healthy adults of a Western population to characterize the ecology of human-associated microbial communities using 454 pyrosequencing and Illumina sequencing [31,32]. However, little is known about the overall structure of microbiota from different body habitats in healthy Chinese individuals. A good definition of commensal microbiota in these habitats and an understanding of the relationship to health are essential in preventing and combating disease.

The purpose of the present study was to characterize the baseline bacterial communities from various human habitats, such as the nasopharynx, saliva, dominant hand and feces, of healthy male and female Chinese junior undergraduates boarding at Zhejiang University using parallel barcoded 454 pyrosequencing targeting the 16S rRNA gene V3 hypervariable region to gain a “whole-body” view [33,34]. The bacterial profiles of these healthy undergraduates, with the same living environmental circumstances and nearly the same living habits, such as diet, rest, exercise, and hygiene in the university, could represent the real healthy microbiota prevailing in Chinese undergraduates, which might be useful for human microbiota-related diseases analyzing the same age range.

## Results and discussion

### Distinct microbial diversity of different healthy human habitats

Determining the role of our microbiota in disease predisposition and pathogenesis will critically depend upon first defining it in healthy states. Previous studies have shown that there are fundamental differences in the composition and structure of bacterial communities in the same habitats within the subjects of different ethnic and racial backgrounds [35,36]. However, most of the studies investigating microbial composition within a specific habitat using deep sequencing analysis have focused on sampling people from the West and some Asian countries [26,27,31,37,38], and little is known about the overall structure and composition of the healthy Chinese microbiota. This imbalance needs to be addressed to more fully understand the interactions between humans and their microbiota. Human microbial communities were strongly affected by external factors, such as lifestyle, dietary patterns, antibiotic usage, and host genotype [16,39-44]. In China, undergraduate students, often boarding at the same university for 4 or 5 years, have adapted to the specific environmental circumstances of the university and formed almost the same lifestyle features, such as diet, rest, exercise, and hygiene after 2 years. Thus, Chinese undergraduate boarders might be an excellent model to explore the representative microbiota of the human body in the same age range. Unlike previous studies, the well-controlled similar living environmental circumstances for these participants could reduce the influence of various environmental factors on the healthy human microbiota and minimize the variations between participants [27,31,32]. The present study characterized the composition and structure of the healthy microbiota of the nasopharynx, saliva, dominant hands and feces from Chinese male and female boarding undergraduates, and established the baselines for human healthy microbiota, which might be useful in understanding the relationship between health and prevailing diseases in the Chinese population in the same age range. Because most of the undergraduates were not married, the vaginal microbiota from female participants was not included, as gynecologic examinations are not included in routine physical examinations for female participants in China. With nearly the same living environments and habits, as mentioned above, the diversity profiles of bacterial communities from these habitats could be considered as representative microbiota of healthy human bodies. Samples were only collected from a single time point, our data are in agreement with the conventional sampling habits in other studies [23,26,45,46]. Recent studies have also shown that within-subject variation over time is consistently lower than between-subject variation, and that the uniqueness of the microbial community in each individual appears to be relatively stable over time (relative to the population as a whole), which suggests that a set of microbiomes in a given healthy habitat can be considered a core microbiome [2,31].

**Figure 1:**
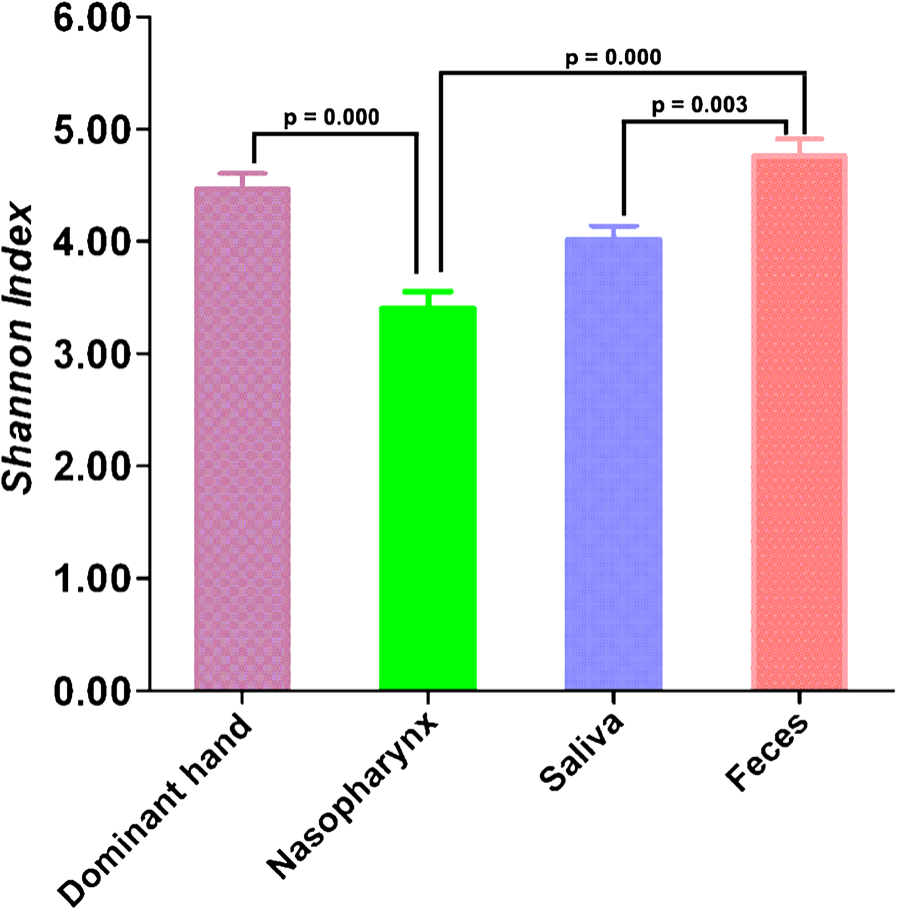
**The Shannon index was used to estimate diversity (i.e., a combined assessment of the number of 3% dissimilar bacterial taxa and their abundance) among nasopharynx, saliva, dominant hand and feces from healthy Chinese undergraduates (Data shown as mean with SEM).**

**Figure 2:**
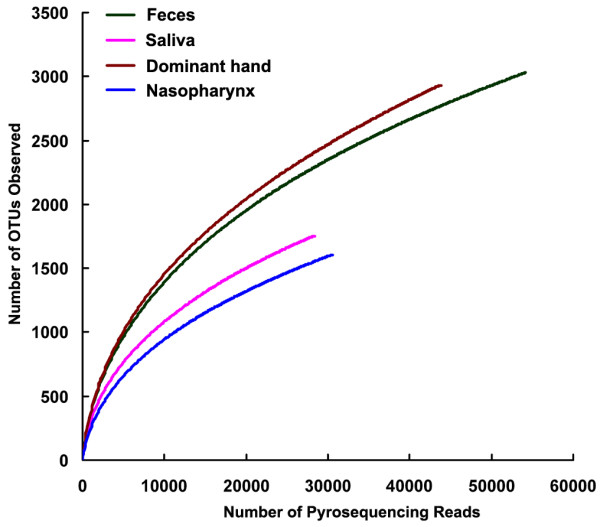
**Rarefaction curves were used to estimate richness (in this case the number of taxa at a 3% dissimilarity level) among nasopharynx, saliva, dominant hand and feces from healthy Chinese undergraduates.** The vertical axis shows the number of OTUs that would be expected to be found after sampling the number of tags or sequences shown on the horizontal axis.

After removing sequences of insufficient quality and sequences that could not be adequately assigned, nearly 156,000 sequences remained from the four human habitats, including 30,521 reads from nasopharynx, 28,309 reads from saliva, 43,708 reads from dominant hands, and 54,179 reads from feces respectively (Table 1). The average length of the sequences was 145 bp after trimming the primers. These high-quality pyrosequencing reads (95.41% of the total sequences) were classified using the RDP Classifier to assign taxonomic classifications to the sequences for ecologic analysis. Although the total number of sequences in each group was no more than in previous studies that mainly focused on a single human habitat [20-24], the average number of nearly 3000 sequences from each habitat in the same participant with a Good’s coverage > 96% could represent the overall structure and composition of human microbiota in the specific healthy habitat. The indices of bacterial richness and diversity of OTUs at a 3% sequence dissimilarity level are summarized in Table 1 and Figure 1, and indicate that the bacterial alpha diversity in these habitats is as follows [though there were dramatic inter-individual variations (Additional file 1: Table S2 and Additional file 2: Figure S1) among participants in each group]: feces > dominant hand > saliva > nasopharynx. However, the established patterns of the bacterial diversity from healthy Chinese undergraduates were not consistent with the results of the HMP, which showed that the oral and fecal communities were especially diverse in terms of community membership, while skin microbiota had less complex alpha diversity [31]. The disparity might be due to less Chinese participant enrollment, fewer sampling sites in each habitat, different sequencing targets, and less sequencing depths in our study. In combination with our previous studies, the diversity of nasopharyngeal microbiota might be as low as vaginal microbiota from healthy Chinese women of reproductive-age [20] when compared with the other three major habitats. In fact, the number of phylotypes detected in a sample, or the number of microorganisms discerned at any given phylogenetic level, was strongly affected by the sequencing depth. With a relatively larger total number of sequences, previous studies have shown that the bacterial diversity in the oral cavity was much more complex than the skin [23,24]. However, Costello *et al*. also claimed that several skin locations harbored more diverse communities than the gastrointestinal tract and oral cavity, which was consistent with the data herein [30]. The richness of total bacterial communities in the habitats was estimated by rarefaction analysis. The trend of the rarefaction curves also confirmed the differences in these habitats mentioned above; however, the unsaturated shapes of the rarefaction curves indicated that bacterial richness of the nasopharyngeal swabs, saliva, palmar swabs and feces was not yet completely sampled (Figure 2).

**Table 1:** Comparison of phylotype coverage and diversity estimation of the 16S rRNA gene libraries at 3% dissimilarity from the pyrosequencing analysis

***Group***	***Reads***	***OTUs***^**1**^	***Good’s***^**2**^	***ACE***	***95% C*. *I *.**		***Chao*1**	***95% C*. *I *.**		***Shannon***	***Simpson***	***Evenness***^**3**^
**Nasopharynx**	30521	998	0.969	2290.3	2142.5	2457.3	1802.0	1622.9	2032.6	4.0960	0.0945	0.0117
**Saliva**	28309	1129	0.962	2431.9	2286.5	2595.7	1907.9	1742.1	2118.6	4.6437	0.0780	0.0131
**Dominant hand**	43708	1768	0.960	3993.1	3796.7	4208.6	3082.0	2853.2	3359.2	5.2870	0.0297	0.0279
**Feces**	54179	1959	0.965	3967.6	3787.5	4165.7	3233.9	3019.2	3492.5	5.4907	0.0197	0.0349

^**1**^The operational taxonomic units (OTUs) were defined with 3% dissimilarity level.

^**2**^The coverage percentage (Good’s), richness estimators (ACE and Chao1) and diversity indices (Shannon and Simpson) were calculated using Good’s method [66] and the mothur program, respectively.

^**3**^The Shannon index of evenness was calculated with the formula E = H/ln(S), where H is the Shannon diversity index and S is the total number of sequences in that group.

**Figure 3:**
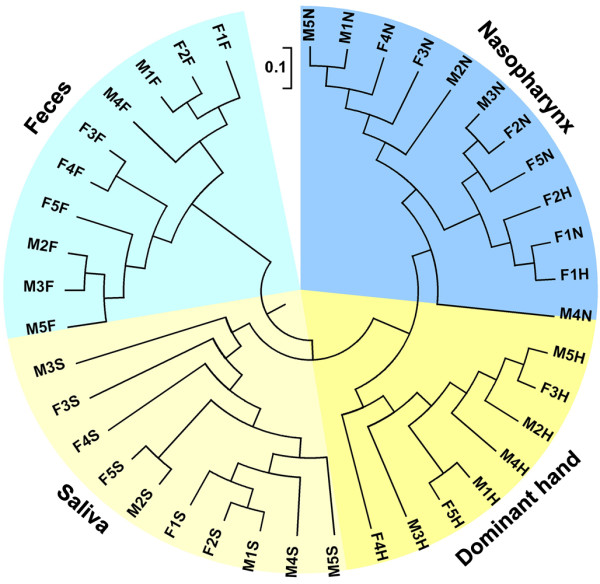
**Differentiation in bacterial communities from all participants (interpersonal variations).** Community differentiation was measured by using the unweighted UniFrac algorithm; the scale bar indicated the distance between clusters in UniFrac units. All of the branch nodes shown here were found to be significant, indicating that bacterial community from each habitat was divided into a distinctive cluster.

**Figure 4:**
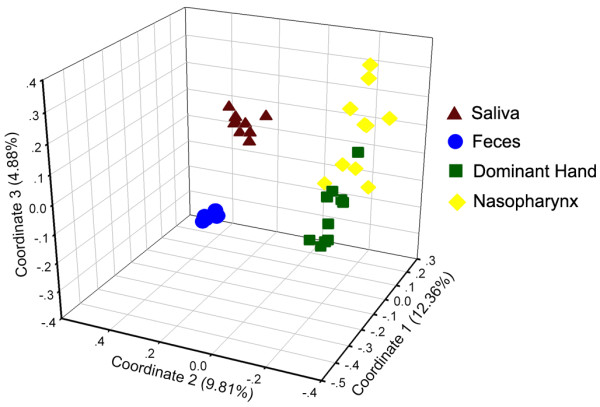
**3D PCoA plots of individual bacterial communities from the four healthy habitats in Chinese undergraduates obtained by mothur program and Sigmaplot.** 3D PCoA plots demonstrating that primary clustering was by anatomical site, with the saliva, feces, dominant hand and nasopharynx separate.

**Figure 5:**
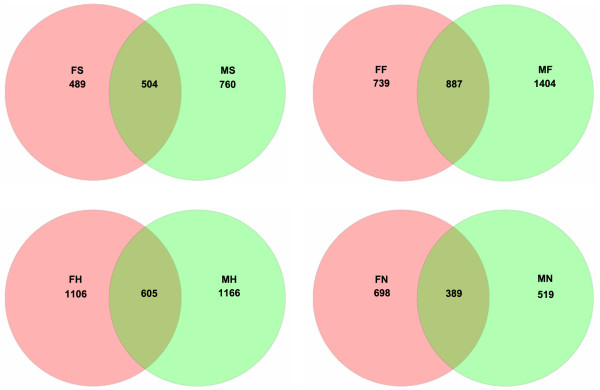
**Venn diagrams illustrating overlap of OTUs by habitat**, **for male and female subjects.**

**Figure 6:**
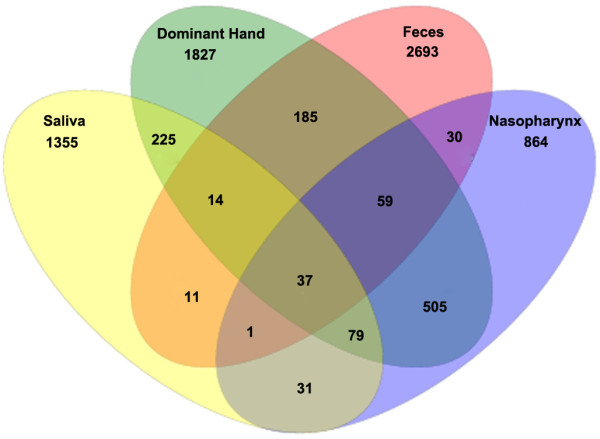
**A Venn diagram illustrating overlap of OTUs for nasopharynx**, **saliva**, **dominant hand and feces from healthy Chinese undergraduates.** A total of 7916 OTUs were detected. Only thirty-seven OTUs were detected in all four habitats.

We assessed the differences in overall bacterial community composition using a phylogeny-based metric (UniFrac) [47]. A relatively small UniFrac distance implied that two communities were compositionally similar, harboring lineages sharing a common evolutionary history. In turn, a large UniFrac distance indicated a quite different structure of microbiota in the habitat. A clear clustering according to anatomic site was observed using unweighted UniFrac, despite significant inter-individual variability (Figure 3). With the same living environmental circumstances and nearly the same living habits for these subjects, each anatomic location within a healthy individual would shape the composition of a microbial community specifically adapted to that site. Principal Coordinates Analysis (PCoA) was performed using weighted, normalized abundance data, demonstrating that primary clustering was by anatomic site, with the saliva, feces, dominant hands, and nasopharynx separate (Figure 4). The data herein were also consistent with the results of a large-scale study from the HMP [30,31]. The current study showed that microbiota from one human habitat was distinct from other human habitats in the same individuals, visually and directly. In spite of significant inter-individual variations, the healthy male and female students still shared a great number of OTUs, and the shared OTUs were represented by the overlapping areas of circles in Venn diagrams, which could be used to understand the relative stable, consistent components across complex microbial assemblages (Figure 5). The results herein indicated that there might be a core microbiome in the specific habitat of the healthy human body. However, the Venn diagrams showed that bacteria from the four different habitats of the healthy human body could not share a distinct core microbiome (only 37 OTUs shared) in each individual (Figure 6), which was consistent with the results of HMP [31].

### Taxonomic analysis of healthy microbiota in different habitats

**Figure 7:**
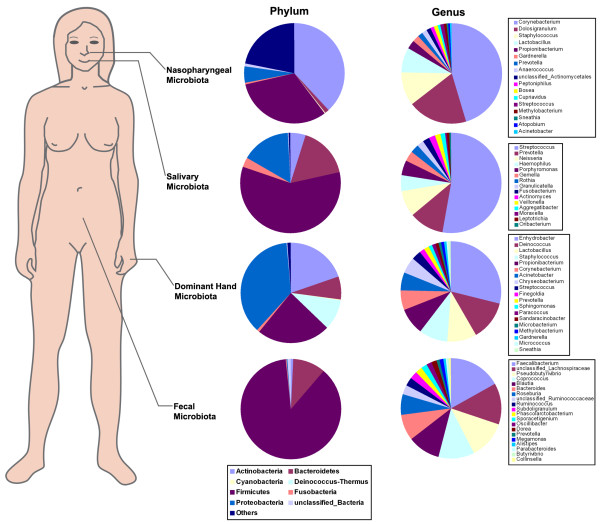
**Compositional differences in the microbiome by anatomical site.** The relative proportion of sequences determined at the phylum and genus level for all four habitats from healthy Chinese undergraduates.

The sequences collected for this study provide an overview of the healthy human microbiota. These sequences could be clustered into 29,887 unique 16S rRNA gene V3 tagged sequences, representing 15 known phyla or candidate divisions and 424 genera (Additional file 3: Table S1). In male or female students, the vast majority of sequences in the human body belonged to one of the five following major phyla: Actinobacteria, Bacteroidetes, Firmicutes, Fusobacteria, and Proteobacteria (Figure 7).

**Figure 8:**
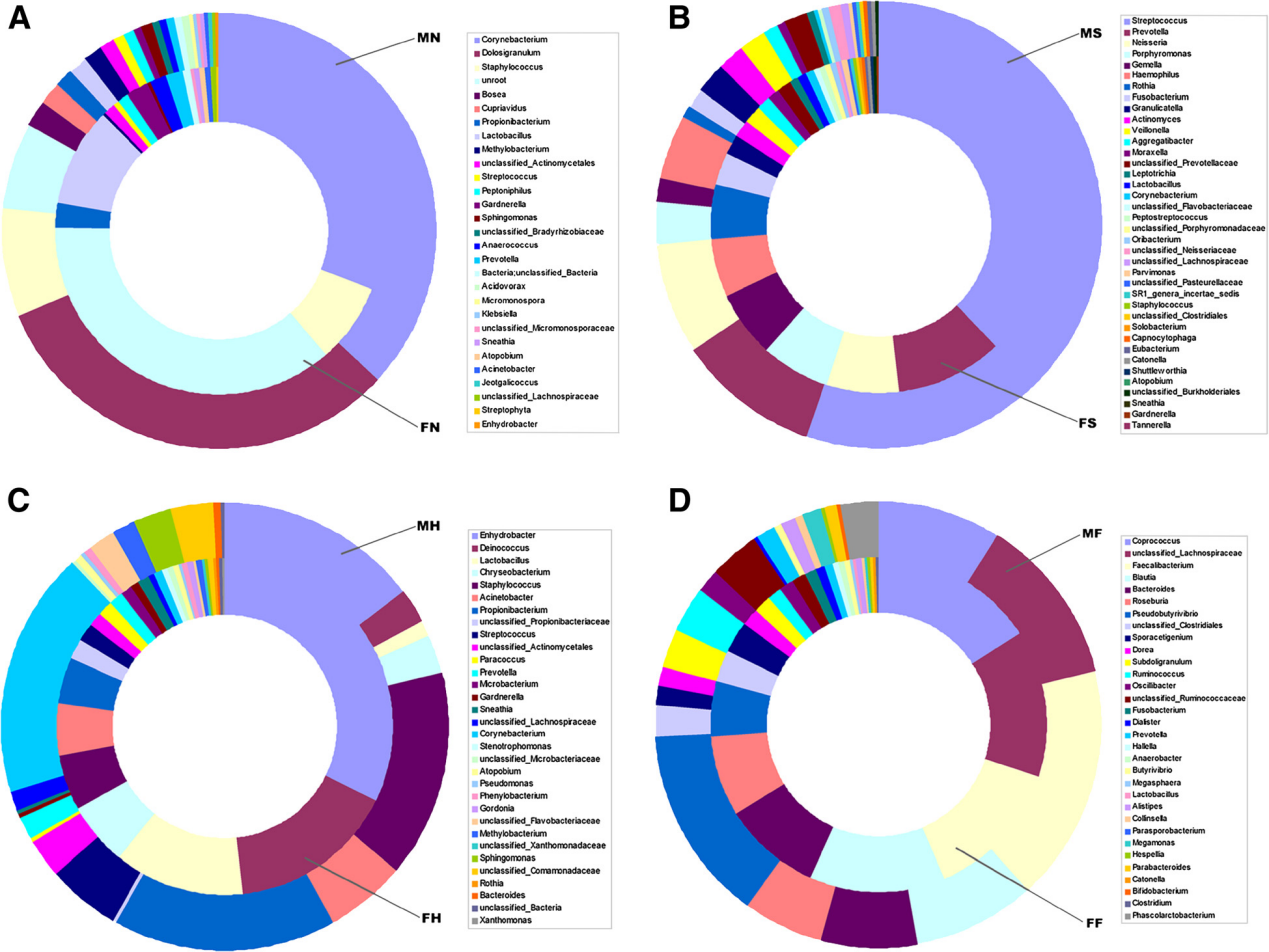
The relative abundance of predominant genera obtained by pyrosequencing from nasopharynx (A), saliva (B), dominant hand (C) and feces (D) of male and female healthy Chinese undergraduates.

In the nasopharynx, 14 phyla, including Actinobacteria, Bacteroidetes, Cyanobacteria, Deinococcus-Thermus, Firmicutes, Fusobacteria, Proteobacteria, Tenericutes, and candidate division TM7 were found. However, most sequences (76.5%) were related to the following four phyla: Actinobacteria (38.1%), Bacteroidetes (1.4%), Firmicutes (31.7%), and Proteobacteria (5.4%), which was consistent with a previous study [48]. The healthy nasopharyngeal microbiota was the only site in which Actinobacteria predominated. It was unexpected that a great number of sequences (22.1%, most often in female students) in the nasopharynx are still unknown. At the genus level, sequences from nasopharyngeal swabs represented 194 different genera (148 genera from females and 132 genera from males). The most frequently detected genera (OTU proportions > 1%) were *Corynebacterium* (32.4%), *Dolosigranulum* (15.3% [male only]), *Staphylococcus* (7.7%), *Lactobacillus* (5.4%), *Propionibacterium* (2.0%), *Gardnerella* (1.5%), unclassified Actinomycetales (1.1%), *Anaerococcus* (1.1%) and *Prevotella* (1.1%) (Figure 8A), which were considered the predominant bacteria, and were also detected using PCR-DGGE and cloning and sequencing techniques. The other 185 genera accounted for the remainder of the genus-level diversity (approximately 32.5% of the total number of sequences), indicative of a low abundance. At this stage, it is difficult to predict the role bacteria present in low numbers, play in nasopharyngeal ecology. However, it would be an oversimplification to neglect their presence. Laufer *et al*. (2011) reported that members of the nasopharyngeal healthy microbiota, i.e., *Corynebacterium*, *Dolosigranulum*, *Staphylococcus*, *Lactococcus*, and *Propionibacterium*, are protective against *Streptococcus pneumoniae* colonization and otitis media [49]. *Streptococcus* load is negatively correlated with *Corynebacterium* and *Dolosigranulum*, suggesting a competitive interaction between this potential otitis media pathogen and these two genera. Surprisingly, the data herein showed that *Dolosigranulum* (Carnobacteriaceae family) was only detected in male participants, and characterized the overall structure and composition of human nasopharyngeal healthy microbiota in a relatively deeper level with high-throughput sequencing techniques. This could be used to explore the relationship between nasopharyngeal microbiota and allergic diseases in the future [50,51].

It is necessary to define the salivary microbiota of the healthy human body before determining the role of oral bacteria in diseases. Nine phyla, including Actinobacteria, Bacteroidetes, Firmicutes, Fusobacteria, Proteobacteria, Spirochaetes, Tenericutes, and two candidate divisions (SR1, TM7), were found [29]. The vast majority of sequences in saliva (99.2%) belonged to one of the following five major phyla: Actinobacteria (4.9%), Bacteroidetes (16.8%), Firmicutes (58.6%), Fusobacteria (3.0%) and Proteobacteria (15.9%) (Figure 7). In fact, the diversity of salivary microbiota was quite different from those in different ages and ethnic groups [23]. When compared with the salivary microbiota from Chinese undergraduates, Bacteroidetes (28.8%), Fusobacteria (5.2%), and Proteobacteria (19.5%) were overrepresented in healthy Chinese children [21], while Firmicutes sequences were more abundant in adult. These disparities might be due to different hygiene habits, different dietary constitution, and different physiologic changes, which were clearly associated with age. When compared with healthy adult Americans reported by Keijser (2008), a higher relative abundance of Bacteroidetes (26.6%) and Proteobacteria (19.9%), and a lower relative abundance of Firmicutes (38.5%) were found in salivary microbiota of Americans, which might be ascribed to different lifestyles and different host genotypes [23]. At the genus level, sequences from saliva represented 105 different genera (88 genera from females and 80 genera from males). The most frequently detected genera in saliva (OTU proportions > 1%) were *Streptococcus* (45.5%), *Prevotella* (10.1%), *Neisseria* (7.4%), *Haemophilus* (5.1%), *Porphyromonas* (4.7%), *Gemella* (3.9%), *Rothia* (3.0%), *Granulicatella* (2.2%), *Fusobacterium* (2.1%), *Actinomyces* (1.8%), *Veillonella* (1.7%) and *Aggregatibacter* (1.3%) (Figure 7 and Figure 8B). The overall taxonomic distribution of the 16S rRNA gene-based amplicons at the genus level mentioned above was in general agreement with previous findings [23], although the relative abundance of several genera was not consistent with the data herein, especially the relative abundance of *Streptococcus*. The higher relative abundance of *Streptococcus*, an acid-producing cariogenic bacterium, was more abundant in the Chinese population. The above-mentioned genera provide a framework for Chinese healthy salivary microbiota diversity and investigations of the environmental, dietary, and genetic factors that influence salivary microbiota in individuals. The continuous flow of saliva made it impossible to obtain a stable salivary microbiota, but only captured the temporal stability of salivary microbiota at a single time point.

In the dominant hands, 14 phyla, including Actinobacteria, Bacteroidetes, Chloroflexi, Cyanobacteria, Deinococcus-Thermus, Firmicutes, Fusobacteria, Proteobacteria, Spirochaetes, Tenericutes, Verrucomicrobia, and three candidate divisions (OP10, SR1, and TM7) were found. Most of the sequences (97.6%) were divided into one of the following five major phyla: Actinobacteria (19.7%), Bacteroidetes (7.5%), Deinococcus-Thermus (9.8%), Firmicutes (23.7%) and Proteobacteria (36.9%) (Figure 7). Interestingly, the healthy skin microbiota from Chinese was the only site in which Proteobacteria predominated when compared with other human habitats. One predominant phylum, Deinococcus-Thermus, was detected in healthy skin microbiota with a relatively high abundance, which has been recognized solely in this habitat. Bacteria from the phylum Deinococcus-Thermus are known for their resistance to extreme stresses, including radiation, oxidation, desiccation, and high temperature. Perhaps due to the special environmental conditions of the hand (e.g., sebum production, salinity, and hydration), Deinococcus-Thermus was found with a higher relative abundance in the skin microbiota. However, the phylum of Deinococcus-Thermus could not be recognized as the predominant bacteria in skin through conventional 16S rRNA gene full-length cloning and sequencing technique [52], which might be ascribed to the methodologic constraints, such as lower resolution [34]. At the genus level, sequences from skin represented 351 different genera (277 genera from females and 262 genera from males). The most frequently detected genera in skin (OTU proportions > 1%) were *Enhydrobacter* (19.8%), *Deinococcus* (8.1%), *Staphylococcus* (7.6%), *Propionibacterium* (7.6%), *Corynebacterium* (6.5%), *Lactobacillus* (6.1%), *Acinetobacter* (4.3%), *Chryseobacterium* (3.8%), *Streptococcus* (2.6%), *Finegoldia* (1.8%), *Sphingomonas* (1.2%), and *Prevotella* (1.1%) (Figure 7 and 8C). Compared with the study conducted by Fierer *et al*. (2008), the taxonomic composition of skin microbiota of healthy Chinese undergraduates was quite different from healthy Americans [24]. In the present study, the genus, *Enhydrobacter* (Proteobacteria phylum), was the most predominant bacteria; however, the role of *Enhydrobacter* on skin microbiota is still unknown. Other genera, as mentioned above, have previously been found to be abundant in other molecular surveys of skin bacteria [24,25,53] and are considered to be common skin residents. A previous study showed that the male and female students harbored significantly different bacterial communities on the hand surfaces [24]. However, similar results between the two genders could not be obtained in Chinese adults. Our current pyrosequencing analysis of bacterial 16S rRNA gene amplicons generated directly from superficial human palms raises the possibility of better understanding of the normal microbial ecology of the skin, and for studying the role of novel microbes or microbial communities.

With respect to the gastrointestinal tract, the composition and structure of the fecal microbiota has been recognized in a number of studies. In the current study, nine phyla, including Actinobacteria, Bacteroidetes, Deinococcus-Thermus, Firmicutes, Fusobacteria, Proteobacteria, Spirochaetes, Tenericutes, and Verrucomicrobia, were found. The two most abundant phyla, Bacteroidetes (10.5%) and Firmicutes (87.1%), constituted > 97% of the total number of sequences and should be considered as the predominant bacteria in this habitat (Figure 7). At the genus level, sequences from feces could be classified as 119 different genera (85 genera from females and 105 genera from males). The most dominant taxonomic groups in fecal microbiota (OTU proportions > 1%) were *Faecalibacterium* (14.8%), unclassified Lachnospiraceae (12.7%), *Coprococcus* (11.9%), *Blautia* (10.6%), *Pseudobutyrivibrio* (9.3%), *Bacteroides* (7.9%), *Roseburia* (6.6%), unclassified Clostridiales (2.5%), unclassified Ruminococcaceae (2.4%), *Ruminococcus* (2.2%), *Subdoligranulum* (2.1%), *Sporacetigenium* (2.1%), *Oscillibacter* (1.6%), *Dorea* (1.5%), *Phascolarctobacterium* (1.4%) and *Prevotella* (1.0%), which accounted for > 90% of the total sequences (Figures 7 and 8D). Except for *Bacteroides* and *Prevotella* (members of the order Bacteroidales), other predominant genera belonged to the order Clostridiales. This community was very similar to that reported for the human ileum and likely represented the normal gastrointestinal microbial population [54]. As shown in previous studies, these resident bacteria constitute a healthy gastrointestinal microbiota and exert a conditioning effect on gastrointestinal homeostasis, delivering regulatory signals to the epithelium and instructing mucosal immune responses [5,8].

There were some limitations to the current study. First, a relatively small number of healthy unmarried Chinese undergraduates participated in the study, which would affect the comprehensive understanding of healthy human microbiota. The microbiota from healthy male and female urogenital tracts of Chinese adults would effectively complete the framework of the human microbiome. Second, only samples from these habitats at a single time point were included; samples from more time points would verify the representative healthy human microbiota. Third, it still could not be ignored the wide variations observed among participants in present study. Living in the similar conditions, the wide OTU differences reported (104–777 in saliva; 219–1215 in feces; 95–1148 in dominant hand; 103–439 in nasopharynx) might be due to the different sequencing depth and different host genotype. A much greater sequencing depth was needed for establishing a database of Chinese healthy microbiota.

## Conclusions

In summary, the framework of microbial communities from four healthy human habitats of the same participants has been established for Chinese undergraduates for the first time. The overall structure of healthy microbiota from these habitats was quite different from each other, which might be due to the specific microenvironments of these anatomic locations. As commensal or symbiotic bacteria, these healthy microbial communities co-exist with their specific habitats in the long-term evolutionary process. Hence, the data herein represent an important step for determining the diversity of the Chinese healthy microbiota, and can be used for additional large-scale studies focusing on the interactions between healthy and disease states for young Chinese adults in the same age range. In fact, the essential roles of microbiota in human health and disease, especially novel microbes, are gaining recognition. With an integrated “whole-body” view, many human complex diseases are associated with microbiota from more than one habitat, such as cardiovascular diseases [55]. The role of microbiota in such human diseases will provide new insights regarding pathogenesis and treatment, and can be utilized as a novel therapeutic target for antibiotics, prebiotics, and probiotics, which indicates that the maintenance or restoration of normal microbiota plays a vital role in human health.

## Methods

### Study population

Ten healthy junior undergraduates 21–24 years of age boarding at the College of Medicine of Zhejiang University, including five male and five female students from two dormitories, were recruited in the present study. Having stayed in the university for > 2 years, these participants were adapted to the same living environmental circumstances, such as the same living spaces, dining hall, and drinking water, and formed nearly the same living habits, such as diet, rest, exercise, and hygiene (including brushing teeth and washing in the morning and evening). Before sampling, the students presented to our hospital in July 2010 for routine medical examinations to confirm each subject’s health status. The following exclusion criteria were followed: body mass index (BMI) > 24 kg/m^2^; hypertension; diabetes mellitus; dyslipidemia; allergic rhinitis; asthma; pharyngitis; dental caries; periodontal disease; various dermatoses; gastrointestinal diseases (diarrhea, constipation, ulcers, bleeding, and tumors); the use of antibiotics, probiotics, prebiotics, or synbiotics in the last 30 days; vegetarian; smoking; alcohol consumption; menstruation at the time of sampling; and known active bacterial, fungal, or viral infections. Informed written consent was obtained from all participants prior to enrollment with approval of the Ethics Committee of the First Affiliated Hospital, College of Medicine of Zhejiang University (Zhejiang province, China).

### Sample collection

After enrollment, specimens (nasopharyngeal swabs, saliva, dominant palmar swabs, and feces) were collected in our hospital from every participant before washing hands, brushing teeth, or having their morning breakfast. Nasopharyngeal specimens were obtained with flexible, sterile, dry swabs, which could reach the posterior nasopharynx easily (approximately 2 inches) and were gently rotated five times around the inside of both nostrils while applying constant pressure. The palmar surfaces of the dominant hands (usually the right hand) were swabbed with cotton-tipped swabs moistened with a solution of 0.15 M NaCl and 0.1% Tween-20. The entire palmar surface was swabbed in two perpendicular directions to ensure that the maximum surface area of the palm was represented in the sample. A 1-ml spontaneous, unstimulated whole saliva specimen was collected into a sterile cryogenic vial (Corning, NY, USA) and fecal samples (300 mg/tube) were collected in sterile tubes. Saliva and fecal samples were put in aliquots before freezing. All samples for bacterial genomic DNA extraction were transferred immediately to the laboratory in an ice box, and stored at −80°C after preparation within 15 min.

### Total bacterial genomic DNA extraction

The bacterial cells retrieved on nasopharyngeal swabs and palmar swabs were submerged in 1 ml of sterile normal saline (prepared with RNase-free H_2_O [pH 7.0]) and vigorously agitated to dislodge cells, while unused swabs served as negative controls, which were handled in the same way. The cells were pelleted by centrifugation (Thermo Electron Corporation, Boston, MA, USA) at full speed (≥ 10,000 g) for 10 min, washed by re-suspending the cells in sterile normal saline, and centrifuged at full speed for 5 min. Then, bacterial DNA was extracted from the swabs using a QIAamp® DNA Mini Kit (QIAGEN, Hilden, Germany) according to the manufacturer’s instructions, with the following minor modification: samples were agitated with 100 mg of zirconium beads (0.1 mm) in a Mini-beadbeater (FastPrep, Thermo Electron Corporation) for 2 min and then incubated at 56°C for 1 h in lysis solution containing proteinase K [20,21]. Bacteria were pelleted from saliva by centrifugation at full speed for 10 min. Then, bacterial genomic DNA was extracted using a QIAamp® DNA Mini Kit with the aforementioned modifications. Stool bacterial genomic DNA was extracted using a QIAamp® DNA Stool Mini Kit (QIAGEN) according to the manufacturer’s instructions [22]. Bacterial genomic DNA was eluted with elution buffer and stored at −20°C for further analysis.

### PCR and pyrosequencing

PCR amplification of the 16S rRNA gene hypervariable V3-region was performed with universal bacterial primers, which correspond to positions 341 to 534 of the conserved *Escherichia coli* 16S rRNA gene sequence. Amplicon pyrosequencing was performed with standard 454/Roche GS-FLX Titanium protocols, where equal molar of PCR product from each sample was pooled. To pool and sort multiple samples in a single 454 GS-FLX run, we used a set of 8-bp barcodes designed according to Fierer *et al*. [20,21,24,56-59]. The main criterion of the barcodes is that the adjoining nucleotides are different because the single nucleotide repeats are the main source of errors in pyrosequencing technology. The forward primer was a fusion of the 454 Life Science adaptor A, the barcode, and the 341F primer (5′-GCCTCCCTCGCGCCATCAG-NNNNNNNN-CCTACGGGAGGCAGCAG-3′). The reverse primer was a fusion of the 454 life science adaptor B, the same barcode with the forward primer, and 534R (5′-GCCTTGCCAGCCCGCTCA-NNNNNNNN-ATTACCGCGG CTGCTGG-3′). The PCR amplicon library was created for each individual DNA sample. The PCRs were carried out in triplicate 50 μL reactions with 0.6 μM each of the primer, 20 ng of template DNA, and 1× PCR reaction buffer, 2.5 U of *pfu* DNA polymerase (MBI, Fermentas, USA). The amplification program consisted of an initial denaturation step at 94°C for 4 min, followed by 25 cycles, where 1 cycle consisted of 94°C for 30 s (denaturation), 55°C for 30 s (annealing) and 72°C for 30 s (extension), and a final extension of 72°C for 10 min. During amplification, negative controls were also performed. Replicate PCR products of the same sample were assembled within a PCR tube. PCR was performed with a PCR thermocycler (Bio-Rad Laboratories, Hercules, California, USA). Prior to sequencing, the DNA concentration of each PCR product was extracted with the MiniElute® Gel Extraction Kit (QIAGEN) and quantified on a NanoDrop ND-1000 spectrophotometer (Thermo Electron Corporation). The products from different samples were mixed at equal ratios for pyrosequencing with the Genome Sequencer FLX (GS-FLX) system (Roche, Basel, Switzerland) at 454 Life Sciences. Emulsion PCR and pyrosequencing were performed according to the manufacturer’s recommendations [60]. Because samples were pooled by equal mass, a variation in the number of sequences recovered from each sample likely reflected slight biases in PCR efficiency among the primer barcodes.

### Bioinformatic analysis

Raw pyrosequencing reads obtained from the sequencer were denoised using the “pre.cluster” command (http://www.mothur.org/wiki/Pre.cluster) in mothur platform (version 1.25.0; http://www.mothur.org) [61] to remove sequences that are likely due to pyrosequencing error [62]. Using mothur implementation of ChimeraSlayer (http://www.mothur.org/wiki/ChimeraSlayer), chimera sequences arising from the PCR amplification were detected and excluded from the denoised sequences [63]. All pyrosequencing reads were filtered using barcode and primer sequences using a combination of tools from mothur and custom Perl scripts. The resulting sequences were further screened and filtered for quality and length. Sequences were included in the subsequent analysis only if the sequences met all four of the following criteria: (1) the sequence carries the correct barcode and exact match to the primer in at least one end; (2) the sequence carries the correct primer sequence at the other end, even though the barcode is absent; (3) the sequence has a length > 160 nucleotides (excluding barcode and primer A sequences) [64]; and (4) the sequence has no ambiguous bases (Ns) and homopolymers longer than 8 nucleotides. A total of 156,717 high-quality pyrosequencing reads were produced according to barcode- and primer-sequence filtering, which were used for the following analysis.

The high-quality pyrosequencing reads were assigned to samples according to barcodes (details shown in Additional file 3: Table S1). Sequences were aligned in accordance with the SILVA alignment [61,65] and clustered into operational taxonomic units (OTUs). The OTUs that reached at a 97% similarity level were used for alpha diversity (Shannon, Simpson, and Evenness), richness (ACE and Chao1), Good’s coverage [66], Venn diagram, and rarefaction curve analysis using the mothur program. Taxonomy-based analyses were performed by classifying each sequence using the Naïve Bayesian Classifier program of the Michigan State University Center for Microbial Ecology Ribosomal Database Project (RDP) database (http://rdp.cme.msu.edu/) [67,68] with a 50% bootstrap score. Phylogenetic beta diversity measures such as unweighted UniFrac distance metrics analysis was performed using OTUs for each sample using the mothur program [47,69], and principal coordinate analysis (PCoA) was conducted according to the distance matrix created by the mothur program and visualized using SigmaPlot.

### Statistical analysis

Statistical analyses were performed using the SPSS Data Analysis Program (version 16.0; SPSS Inc., Chicago, IL, USA) with a 2-tailed *t*-test or One-Way ANOVA. All tests for significance were two-sided, and *p* values < 0.05 were considered statistically significant.

### Accession numbers

The high-quality sequence data from this study has been deposited in the GenBank Sequence Read Archive with accession number SRP016050.

## Competing interests

The authors declare no competing interests.

## Authors’ contributions

Conceived and designed the experiments: ZL XL KEN CX LL. Performed the experiments: ZL XL YL LY YW CX. Analyzed the data: ZL XL YW KEN CX LL. Contributed reagents/materials/analysis tools: ZL XL YW CX LL. Wrote the paper: ZL XL KEN CX LL. All authors read and approved the final manuscript.

## Supplementary Material

Additional file 1: Table S2 Comparison of phylotypes coverage and diversity estimation of the 16S rRNA gene libraries for individuals at 3% dissimilarity from the pyrosequencing analysis. Click here for file

Additional file 2: Figure S1 Rarefaction curves were used to estimate richness (in this case the number of taxa at a 3% dissimilarity level) among individuals. The vertical axis shows the number of OTUs that would be expected to be found after sampling the number of tags or sequences shown on the horizontal axis. Click here for file

Additional file 3: Table S1 All genera assigned using the RDP-classifier with at least 50% as bootstrap support). Click here for file

## References

[B1] Dethlefsen L McFall-Ngai M Relman DA An ecological and evolutionary perspective on human-microbe mutualism and disease Nature 2007 449 7164 811 818 17943117 10.1038/nature06245 PMC9464033

[B2] Turnbaugh PJ Ley RE Hamady M Fraser-Liggett CM Knight R Gordon JI The human microbiome project Nature 2007 449 7164 804 810 17943116 10.1038/nature06244 PMC3709439

[B3] Gill SR Pop M Deboy RT Eckburg PB Turnbaugh PJ Samuel BS Gordon JI Relman DA Fraser-Liggett CM Nelson KE Metagenomic analysis of the human distal gut microbiome Science 2006 312 5778 1355 1359 16741115 10.1126/science.1124234 PMC3027896

[B4] O’Hara AM Shanahan F The gut flora as a forgotten organ EMBO Rep 2006 7 7 688 693 16819463 10.1038/sj.embor.7400731 PMC1500832

[B5] Cash HL Whitham CV Behrendt CL Hooper LV Symbiotic bacteria direct expression of an intestinal bactericidal lectin Science 2006 313 5790 1126 1130 16931762 10.1126/science.1127119 PMC2716667

[B6] Ley RE Peterson DA Gordon JI Ecological and evolutionary forces shaping microbial diversity in the human intestine Cell 2006 124 4 837 848 16497592 10.1016/j.cell.2006.02.017

[B7] Ley RE Turnbaugh PJ Klein S Gordon JI Microbial ecology: human gut microbes associated with obesity Nature 2006 444 7122 1022 1023 17183309 10.1038/4441022a

[B8] Mazmanian SK Liu CH Tzianabos AO Kasper DL An immunomodulatory molecule of symbiotic bacteria directs maturation of the host immune system Cell 2005 122 1 107 118 16009137 10.1016/j.cell.2005.05.007

[B9] Turnbaugh PJ Ley RE Mahowald MA Magrini V Mardis ER Gordon JI An obesity-associated gut microbiome with increased capacity for energy harvest Nature 2006 444 7122 1027 1031 17183312 10.1038/nature05414

[B10] Ichinohe T Pang IK Kumamoto Y Peaper DR Ho JH Murray TS Iwasaki A Microbiota regulates immune defense against respiratory tract influenza A virus infection Proc Natl Acad Sci USA 2011 108 13 5354 5359 21402903 10.1073/pnas.1019378108 PMC3069176

[B11] Grice EA Segre JA The skin microbiome Nat Rev Microbiol 2011 9 4 244 253 21407241 10.1038/nrmicro2537 PMC3535073

[B12] Wanke I Steffen H Christ C Krismer B Gotz F Peschel A Schaller M Schittek B Skin commensals amplify the innate immune response to pathogens by activation of distinct signaling pathways J Invest Dermatol 2011 131 2 382 390 21048787 10.1038/jid.2010.328

[B13] Naik S Bouladoux N Wilhelm C Molloy MJ Salcedo R Kastenmuller W Deming C Quinones M Koo L Conlan S Compartmentalized control of skin immunity by resident commensals Science 2012 337 6098 1115 1119 22837383 10.1126/science.1225152 PMC3513834

[B14] Maynard CL Elson CO Hatton RD Weaver CT Reciprocal interactions of the intestinal microbiota and immune system Nature 2012 489 7415 231 241 22972296 10.1038/nature11551 PMC4492337

[B15] Kirjavainen PV Pautler S Baroja ML Anukam K Crowley K Carter K Reid G Abnormal immunological profile and vaginal microbiota in women prone to urinary tract infections Clin Vaccine Immunol 2009 16 1 29 36 19020112 10.1128/CVI.00323-08 PMC2620669

[B16] Turnbaugh PJ Hamady M Yatsunenko T Cantarel BL Duncan A Ley RE Sogin ML Jones WJ Roe BA Affourtit JP A core gut microbiome in obese and lean twins Nature 2009 457 7228 480 484 19043404 10.1038/nature07540 PMC2677729

[B17] Frank DN St Amand AL Feldman RA Boedeker EC Harpaz N Pace NR Molecular-phylogenetic characterization of microbial community imbalances in human inflammatory bowel diseases Proc Natl Acad Sci USA 2007 104 34 13780 13785 17699621 10.1073/pnas.0706625104 PMC1959459

[B18] Wen L Ley RE Volchkov PY Stranges PB Avanesyan L Stonebraker AC Hu C Wong FS Szot GL Bluestone JA Innate immunity and intestinal microbiota in the development of Type 1 diabetes Nature 2008 455 7216 1109 1113 18806780 10.1038/nature07336 PMC2574766

[B19] Ordovas JM Mooser V Metagenomics: the role of the microbiome in cardiovascular diseases Curr Opin Lipidol 2006 17 2 157 161 16531752 10.1097/01.mol.0000217897.75068.ba

[B20] Ling Z Kong J Liu F Zhu H Chen X Wang Y Li L Nelson KE Xia Y Xiang C Molecular analysis of the diversity of vaginal microbiota associated with bacterial vaginosis BMC Genomics 2010 11 488 20819230 10.1186/1471-2164-11-488 PMC2996984

[B21] Ling Z Kong J Jia P Wei C Wang Y Pan Z Huang W Li L Chen H Xiang C Analysis of oral microbiota in children with dental caries by PCR-DGGE and barcoded pyrosequencing Microb Ecol 2010 60 3 677 690 20614117 10.1007/s00248-010-9712-8

[B22] Chen W Liu F Ling Z Tong X Xiang C Human intestinal lumen and mucosa-associated microbiota in patients with colorectal cancer PLoS One 2012 7 6 e39743 22761885 10.1371/journal.pone.0039743 PMC3386193

[B23] Keijser BJ Zaura E Huse SM van der Vossen JM Schuren FH Montijn RC ten Cate JM Crielaard W Pyrosequencing analysis of the oral microflora of healthy adults J Dent Res 2008 87 11 1016 1020 18946007 10.1177/154405910808701104

[B24] Fierer N Hamady M Lauber CL Knight R The influence of sex, handedness, and washing on the diversity of hand surface bacteria Proc Natl Acad Sci USA 2008 105 46 17994 17999 19004758 10.1073/pnas.0807920105 PMC2584711

[B25] Gao Z Tseng CH Pei Z Blaser MJ Molecular analysis of human forearm superficial skin bacterial biota Proc Natl Acad Sci USA 2007 104 8 2927 2932 17293459 10.1073/pnas.0607077104 PMC1815283

[B26] Li M Wang B Zhang M Rantalainen M Wang S Zhou H Zhang Y Shen J Pang X Wei H Symbiotic gut microbes modulate human metabolic phenotypes Proc Natl Acad Sci USA 2008 105 6 2117 2122 18252821 10.1073/pnas.0712038105 PMC2538887

[B27] Qin J Li R Raes J Arumugam M Burgdorf KS Manichanh C Nielsen T Pons N Levenez F Yamada T A human gut microbial gene catalogue established by metagenomic sequencing Nature 2010 464 7285 59 65 20203603 10.1038/nature08821 PMC3779803

[B28] Hilty M Qi W Brugger SD Frei L Agyeman P Frey PM Aebi S Muhlemann K Nasopharyngeal microbiota in infants with acute otitis media J Infect Dis 2012 205 7 1048 1055 22351941 10.1093/infdis/jis024 PMC7107284

[B29] Lazarevic V Whiteson K Hernandez D Francois P Schrenzel J Study of inter- and intra-individual variations in the salivary microbiota BMC Genomics 2010 11 523 20920195 10.1186/1471-2164-11-523 PMC2997015

[B30] Costello EK Lauber CL Hamady M Fierer N Gordon JI Knight R Bacterial community variation in human body habitats across space and time Science 2009 326 5960 1694 1697 19892944 10.1126/science.1177486 PMC3602444

[B31] The Human Microbiome Project Consortium Structure, function and diversity of the healthy human microbiome Nature 2012 486 7402 207 214 22699609 10.1038/nature11234 PMC3564958

[B32] The Human Microbiome Project Consortium A framework for human microbiome research Nature 2012 486 7402 215 221 22699610 10.1038/nature11209 PMC3377744

[B33] Wang T Cai G Qiu Y Fei N Zhang M Pang X Jia W Cai S Zhao L Structural segregation of gut microbiota between colorectal cancer patients and healthy volunteers ISME J 2012 6 2 320 329 21850056 10.1038/ismej.2011.109 PMC3260502

[B34] Huse SM Dethlefsen L Huber JA Mark Welch D Relman DA Sogin ML Exploring microbial diversity and taxonomy using SSU rRNA hypervariable tag sequencing PLoS Genet 2008 4 11 e1000255 19023400 10.1371/journal.pgen.1000255 PMC2577301

[B35] Zhou X Brown CJ Abdo Z Davis CC Hansmann MA Joyce P Foster JA Forney LJ Differences in the composition of vaginal microbial communities found in healthy Caucasian and black women ISME J 2007 1 2 121 133 18043622 10.1038/ismej.2007.12

[B36] Zhou X Hansmann MA Davis CC Suzuki H Brown CJ Schutte U Pierson JD Forney LJ The vaginal bacterial communities of Japanese women resemble those of women in other racial groups FEMS Immunol Med Microbiol 2010 58 2 169 181 19912342 10.1111/j.1574-695X.2009.00618.x PMC2868947

[B37] Nam YD Jung MJ Roh SW Kim MS Bae JW Comparative analysis of Korean human gut microbiota by barcoded pyrosequencing PLoS One 2011 6 7 e22109 21829445 10.1371/journal.pone.0022109 PMC3146482

[B38] Kurokawa K Itoh T Kuwahara T Oshima K Toh H Toyoda A Takami H Morita H Sharma VK Srivastava TP Comparative metagenomics revealed commonly enriched gene sets in human gut microbiomes DNA Res 2007 14 4 169 181 17916580 10.1093/dnares/dsm018 PMC2533590

[B39] Dicksved J Floistrup H Bergstrom A Rosenquist M Pershagen G Scheynius A Roos S Alm JS Engstrand L Braun-Fahrlander C Molecular fingerprinting of the fecal microbiota of children raised according to different lifestyles Appl Environ Microbiol 2007 73 7 2284 2289 17293501 10.1128/AEM.02223-06 PMC1855685

[B40] Lay C Rigottier-Gois L Holmstrom K Rajilic M Vaughan EE de Vos WM Collins MD Thiel R Namsolleck P Blaut M Colonic microbiota signatures across five northern European countries Appl Environ Microbiol 2005 71 7 4153 4155 16000838 10.1128/AEM.71.7.4153-4155.2005 PMC1169042

[B41] Mueller S Saunier K Hanisch C Norin E Alm L Midtvedt T Cresci A Silvi S Orpianesi C Verdenelli MC Differences in fecal microbiota in different European study populations in relation to age, gender, and country: a cross-sectional study Appl Environ Microbiol 2006 72 2 1027 1033 16461645 10.1128/AEM.72.2.1027-1033.2006 PMC1392899

[B42] Hayashi H Sakamoto M Benno Y Fecal microbial diversity in a strict vegetarian as determined by molecular analysis and cultivation Microbiol Immunol 2002 46 12 819 831 12597356 10.1111/j.1348-0421.2002.tb02769.x

[B43] Jernberg C Lofmark S Edlund C Jansson JK Long-term ecological impacts of antibiotic administration on the human intestinal microbiota ISME J 2007 1 1 56 66 18043614 10.1038/ismej.2007.3

[B44] Dethlefsen L Huse S Sogin ML Relman DA The pervasive effects of an antibiotic on the human gut microbiota, as revealed by deep 16S rRNA sequencing PLoS Biol 2008 6 11 e280 19018661 10.1371/journal.pbio.0060280 PMC2586385

[B45] Grice EA Kong HH Renaud G Young AC Bouffard GG Blakesley RW Wolfsberg TG Turner ML Segre JA A diversity profile of the human skin microbiota Genome Res 2008 18 7 1043 1050 18502944 10.1101/gr.075549.107 PMC2493393

[B46] Caporaso JG Lauber CL Costello EK Berg-Lyons D Gonzalez A Stombaugh J Knights D Gajer P Ravel J Fierer N Moving pictures of the human microbiome Genome Biol 2011 12 5 R50 21624126 10.1186/gb-2011-12-5-r50 PMC3271711

[B47] Lozupone C Knight R UniFrac: a new phylogenetic method for comparing microbial communities Appl Environ Microbiol 2005 71 12 8228 8235 16332807 10.1128/AEM.71.12.8228-8235.2005 PMC1317376

[B48] Frank DN Feazel LM Bessesen MT Price CS Janoff EN Pace NR The human nasal microbiota and Staphylococcus aureus carriage PLoS One 2010 5 5 e10598 20498722 10.1371/journal.pone.0010598 PMC2871794

[B49] Laufer AS Metlay JP Gent JF Fennie KP Kong Y Pettigrew MM Microbial communities of the upper respiratory tract and otitis media in children MBio 2011 2 1 e00245-10 21285435 10.1128/mBio.00245-10 PMC3031303

[B50] Huffnagle GB The microbiota and allergies/asthma PLoS Pathog 2010 6 5 e1000549 20523892 10.1371/journal.ppat.1000549 PMC2877736

[B51] Ege MJ Mayer M Normand AC Genuneit J Cookson WO Braun-Fahrlander C Heederik D Piarroux R von Mutius E Exposure to environmental microorganisms and childhood asthma N Engl J Med 2011 364 8 701 709 21345099 10.1056/NEJMoa1007302

[B52] Staudinger T Pipal A Redl B Molecular analysis of the prevalent microbiota of human male and female forehead skin compared to forearm skin and the influence of make-up J Appl Microbiol 2011 110 6 1381 1389 21362117 10.1111/j.1365-2672.2011.04991.x

[B53] Dekio I Hayashi H Sakamoto M Kitahara M Nishikawa T Suematsu M Benno Y Detection of potentially novel bacterial components of the human skin microbiota using culture-independent molecular profiling J Med Microbiol 2005 54 Pt 12 1231 1238 16278439 10.1099/jmm.0.46075-0

[B54] Hartman AL Lough DM Barupal DK Fiehn O Fishbein T Zasloff M Eisen JA Human gut microbiome adopts an alternative state following small bowel transplantation Proc Natl Acad Sci USA 2009 106 40 17187 17192 19805153 10.1073/pnas.0904847106 PMC2746123

[B55] Koren O Spor A Felin J Fak F Stombaugh J Tremaroli V Behre CJ Knight R Fagerberg B Ley RE Human oral, gut, and plaque microbiota in patients with atherosclerosis Proc Natl Acad Sci USA 2011 108 Suppl 1 4592 4598 20937873 10.1073/pnas.1011383107 PMC3063583

[B56] Parameswaran P Jalili R Tao L Shokralla S Gharizadeh B Ronaghi M Fire AZ A pyrosequencing-tailored nucleotide barcode design unveils opportunities for large-scale sample multiplexing Nucleic Acids Res 2007 35 19 e130 17932070 10.1093/nar/gkm760 PMC2095802

[B57] Hamady M Walker JJ Harris JK Gold NJ Knight R Error-correcting barcoded primers for pyrosequencing hundreds of samples in multiplex Nat Methods 2008 5 3 235 237 18264105 10.1038/nmeth.1184 PMC3439997

[B58] Ling Z Liu X Wang Y Li L Xiang C Pyrosequencing analysis of the salivary microbiota of healthy Chinese children and adults Microb Ecol 2013 65 2 487 495 22968328 10.1007/s00248-012-0123-x

[B59] Ling Z Liu X Chen W Luo Y Yuan L Xia Y Nelson KE Huang S Zhang S Wang Y The restoration of the vaginal microbiota after treatment for bacterial vaginosis with metronidazole or probiotics Microb Ecol 2013 65 3 773 780 23250116 10.1007/s00248-012-0154-3

[B60] Margulies M Egholm M Altman WE Attiya S Bader JS Bemben LA Berka J Braverman MS Chen YJ Chen Z Genome sequencing in microfabricated high-density picolitre reactors Nature 2005 437 7057 376 380 16056220 10.1038/nature03959 PMC1464427

[B61] Schloss PD Westcott SL Ryabin T Hall JR Hartmann M Hollister EB Lesniewski RA Oakley BB Parks DH Robinson CJ Introducing mothur: open-source, platform-independent, community-supported software for describing and comparing microbial communities Appl Environ Microbiol 2009 75 23 7537 7541 19801464 10.1128/AEM.01541-09 PMC2786419

[B62] Roeselers G Mittge EK Stephens WZ Parichy DM Cavanaugh CM Guillemin K Rawls JF Evidence for a core gut microbiota in the zebrafish ISME J 2011 5 10 1595 1608 21472014 10.1038/ismej.2011.38 PMC3176511

[B63] Haas BJ Gevers D Earl AM Feldgarden M Ward DV Giannoukos G Ciulla D Tabbaa D Highlander SK Sodergren E Chimeric 16S rRNA sequence formation and detection in Sanger and 454-pyrosequenced PCR amplicons Genome Res 2011 21 3 494 504 21212162 10.1101/gr.112730.110 PMC3044863

[B64] Roh SW Kim KH Nam YD Chang HW Park EJ Bae JW Investigation of archaeal and bacterial diversity in fermented seafood using barcoded pyrosequencing ISME J 2010 4 1 1 16 19587773 10.1038/ismej.2009.83

[B65] Pruesse E Quast C Knittel K Fuchs BM Ludwig W Peplies J Glockner FO SILVA: a comprehensive online resource for quality checked and aligned ribosomal RNA sequence data compatible with ARB Nucleic Acids Res 2007 35 21 7188 7196 17947321 10.1093/nar/gkm864 PMC2175337

[B66] Good IJ The population frequencies of species and the estimation of population parameters Biometrika 1953 40 237 264

[B67] Wang Q Garrity GM Tiedje JM Cole JR Naive Bayesian classifier for rapid assignment of rRNA sequences into the new bacterial taxonomy Appl Environ Microbiol 2007 73 16 5261 5267 17586664 10.1128/AEM.00062-07 PMC1950982

[B68] Rosen GL Reichenberger ER Rosenfeld AM NBC: the Naive Bayes Classification tool webserver for taxonomic classification of metagenomic reads Bioinformatics 2011 27 1 127 129 21062764 10.1093/bioinformatics/btq619 PMC3008645

[B69] Lozupone C Hamady M Knight R UniFrac–an online tool for comparing microbial community diversity in a phylogenetic context BMC Bioinforma 2006 7 371 10.1186/1471-2105-7-371 PMC1564154 16893466

