# Supplementary material for: Pyrosequencing analysis of the human microbiota of healthy Chinese undergraduates
Source: BMC Genomics. 2013 Jun 10;14:390. doi: 10.1186/1471-2164-14-390 (PMC3685588; doi:10.1186/1471-2164-14-390)
Supplement: Additional file 1: Table S2 — Comparison of phylotypes coverage and diversity estimation of the 16S rRNA gene libraries for individuals at 3% dissimilarity from the pyrosequencing analysis. [file 1471-2164-14-390-S1.doc]

**Table S2** Comparison of phylotypes coverage and diversity estimation of the 16S rRNA gene libraries for individuals at 3% dissimilarity from the pyrosequencing analysis

| **Sample** | **Reads** | **OTUs** | **ACE** | **Chao1** | **Shannon** | **Good’s Coverage** |
| --- | --- | --- | --- | --- | --- | --- |
| F1s | 2067 | 382 | 915.69 | 724.16 | 4.65552 | 90.4209% |
| F2s | 4456 | 487 | 1037.91 | 904.39 | 4.19073 | 94.8833% |
| F3s | 386 | 104 | 295.87 | 215.24 | 3.39721 | 83.9378% |
| F4s | 1155 | 222 | 738.50 | 461.57 | 3.81384 | 88.7446% |
| F5s | 1795 | 280 | 592.90 | 473.72 | 4.17830 | 92.5348% |
| M1s | 5225 | 541 | 1070.00 | 821.78 | 4.23145 | 95.3876% |
| M2s | 10728 | 777 | 1687.57 | 1374.38 | 3.93251 | 96.7095% |
| M3s | 528 | 150 | 619.86 | 365.53 | 4.25725 | 82.7652% |
| M4s | 1154 | 227 | 625.24 | 465.31 | 3.98825 | 89.2548% |
| M5s | 815 | 176 | 555.76 | 378.22 | 3.55147 | 87.1166% |
| F1f | 3353 | 672 | 1569.21 | 1237.00 | 5.37259 | 89.8598% |
| F2f | 5847 | 813 | 1902.60 | 1465.92 | 5.17246 | 93.1931% |
| F3f | 1580 | 219 | 760.16 | 531.63 | 4.03423 | 92.2152% |
| F4f | 2400 | 281 | 799.69 | 506.21 | 4.05467 | 93.9167% |
| F5f | 2907 | 539 | 1137.21 | 912.62 | 5.02503 | 91.2625% |
| M1f | 19080 | 1215 | 2825.40 | 1999.86 | 4.51687 | 97.0755% |
| M2f | 9074 | 762 | 1549.87 | 1264.55 | 4.75123 | 96.2641% |
| M3f | 2344 | 404 | 1058.86 | 744.85 | 4.71713 | 90.9983% |
| M4f | 4849 | 789 | 1859.60 | 1441.92 | 5.30196 | 91.7921% |
| M5f | 2745 | 411 | 1217.39 | 882.75 | 4.70700 | 91.9126% |
| F1h | 465 | 171 | 508.25 | 335.16 | 4.44412 | 77.8495% |
| F2h | 4124 | 538 | 1327.37 | 1060.22 | 4.50718 | 93.5257% |
| F3h | 20030 | 1148 | 2429.29 | 1991.03 | 4.21037 | 97.4289% |
| F4h | 313 | 95 | 321.07 | 183.67 | 3.80508 | 81.7891% |
| F5h | 2608 | 392 | 916.02 | 717.05 | 4.49118 | 92.5997% |
| M1h | 2667 | 390 | 943.03 | 716.23 | 4.41432 | 92.5009% |
| M2h | 2862 | 464 | 1299.33 | 833.13 | 4.57025 | 91.5444% |
| M3h | 727 | 184 | 690.52 | 454.26 | 4.12010 | 84.5942% |
| M4h | 5020 | 636 | 1555.57 | 1158.78 | 4.81383 | 93.7450% |
| M5h | 4892 | 802 | 2165.64 | 1521.33 | 5.37178 | 91.4963% |
| F1n | 2781 | 296 | 779.61 | 691.72 | 4.03695 | 94.5343% |
| F2n | 5275 | 390 | 1122.18 | 775.00 | 2.51013 | 96.0190% |
| F3n | 4256 | 317 | 772.93 | 635.49 | 2.69426 | 96.0996% |
| F4n | 3773 | 333 | 807.31 | 596.37 | 3.55224 | 95.6003% |
| F5n | 715 | 149 | 388.39 | 307.45 | 3.87001 | 88.2517% |
| M1n | 2913 | 251 | 658.81 | 461.55 | 3.24728 | 95.6402% |
| M2n | 412 | 103 | 187.28 | 179.00 | 3.49531 | 86.1650% |
| M3n | 3474 | 301 | 602.84 | 479.22 | 3.47461 | 96.1428% |
| M4n | 1418 | 200 | 762.08 | 502.88 | 3.66241 | 91.1142% |
| M5n | 5504 | 439 | 1006.58 | 832.56 | 3.51247 | 96.0756% |

Note: M: male; F: female; s: saliva; f: feces; n: nasopharynx; h: dominant hand.
