# Supplementary figures and images for: Pyrosequencing analysis of the human microbiota of healthy Chinese undergraduates
Source: BMC Genomics. 2013 Jun 10;14:390. doi: 10.1186/1471-2164-14-390 (PMC3685588; doi:10.1186/1471-2164-14-390)

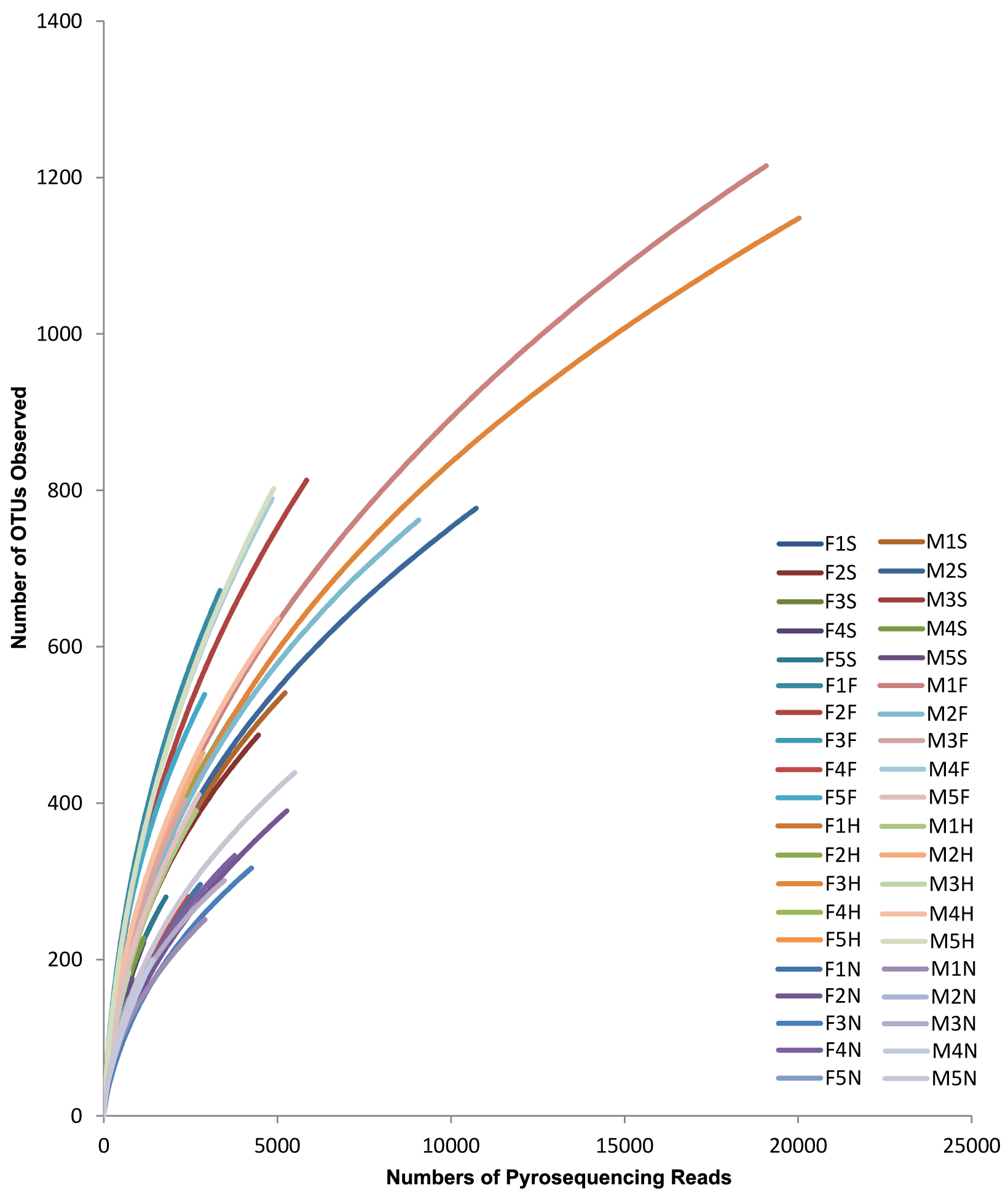

Supplement: Additional file 2: Figure S1 — Rarefaction curves were used to estimate richness (in this case the number of taxa at a 3% dissimilarity level) among individuals. The vertical axis shows the number of OTUs that would be expected to be found after sampling the number of tags or sequences shown on the horizontal axis. [file 1471-2164-14-390-S2.tiff]
